# Egg Intake and Dietary Quality among Overweight and Obese Mexican-American Postpartum Women

**DOI:** 10.3390/nu7105402

**Published:** 2015-10-02

**Authors:** Sonia Vega-López, Giselle A. P. Pignotti, Michael Todd, Colleen Keller

**Affiliations:** 1 School of Nutrition and Health Promotion, Arizona State University, 500 North 3rd Street, Phoenix, AZ 85004, USA; 2 Southwestern Interdisciplinary Research Center, Arizona State University, 411 North Central Avenue, Suite #720, Phoenix, AZ 85004, USA; colleen.keller@asu.edu; 3 Department of Nutrition, Food Science and Packaging, San Jose State University, One Washington Square, San Jose, CA 95192-0058, USA; giselle.pignotti@sjsu.edu; 4 College of Nursing and Health Innovation, Arizona State University, 500 North 3rd Street, Phoenix, AZ 85004, USA; mike.todd@asu.edu

**Keywords:** diet, eggs, healthy eating index, Hispanic women, nutrient intake

## Abstract

Despite their low cost and high nutrient density, the contribution of eggs to nutrient intake and dietary quality among Mexican-American postpartum women has not been evaluated. Nutrient intake and dietary quality, as assessed by the Healthy Eating Index 2010 (HEI-2010), were measured in habitually sedentary overweight/obese (body mass index (BMI) = 29.7 ± 3.5 kg/m^2^) Mexican-American postpartum women (28 ± 6 years) and compared between egg consumers (*n* = 82; any egg intake reported in at least one of three 24-h dietary recalls) and non-consumers (*n* = 57). Egg consumers had greater intake of energy (+808 kJ (193 kcal) or 14%; *p* = 0.033), protein (+9 g or 17%; *p* = 0.031), total fat (+9 g or 19%; *p* = 0.039), monounsaturated fat (+4 g or 24%; *p* = 0.020), and several micronutrients than non-consumers. Regarding HEI-2010 scores, egg consumers had a greater total protein foods score than non-consumers (4.7 ± 0.7 *vs.* 4.3 ± 1.0; *p* = 0.004), and trends for greater total fruit (2.4 ± 1.8 *vs.* 1.9 ± 1.7; *p* = 0.070) and the total composite HEI-2010 score (56.4 ± 12.6 *vs.* 52.3 ± 14.4; *p* = 0.082). Findings suggest that egg intake could contribute to greater nutrient intake and improved dietary quality among postpartum Mexican-American women. Because of greater energy intake among egg consumers, recommendations for overweight/obese individuals should include avoiding excessive energy intake and incorporating eggs to a nutrient-dense, fiber-rich dietary pattern.

## 1. Introduction

Hispanics, the largest minority group in the United States (U.S.) [1], have a higher prevalence of obesity and cardiometabolic disease risk factors relative to other ethnic groups, disproportionately increasing their risk for chronic conditions such as cardiovascular disease and diabetes [2,3]. Among women of reproductive age, excess weight gain during pregnancy and failure to lose weight postpartum have been associated with long-term obesity and further risk for chronic diseases [4]. This is of particular concern for postpartum Hispanic women due to higher pre-pregnancy obesity rates [5] and the presence of many contributors to excessive weight retention after childbirth [6].

Several dietary factors are essential for the prevention and management of chronic diseases. Studies have shown that a higher dietary quality is inversely related to chronic disease risk factors such as waist circumference, low density lipoprotein (LDL)-cholesterol, insulin and C-reactive protein (CRP) [7,8]. Despite the known benefits of consuming adequate diets, available surveillance data on dietary composition suggest that, as for other ethnic groups, the diet of Mexican-American adults is in need for improvement, as indicated by reports of high intake of solid fats, added sugars, and sugar-sweetened beverages, and low intake of vitamins D and E, calcium and potassium, whole grains, dairy products, dark greens, and highly colored vegetables [9,10,11,12,13].

Due to their cholesterol content, the contribution of eggs to a healthful dietary pattern continues to be controversial [14,15,16,17]. Whereas some studies have identified eggs as part of a “healthful” or “prudent” dietary pattern with greater Healthy Eating Index (HEI) scores [18,19], others have identified eggs as components of dietary patterns associated with greater risk for adverse outcomes including overweight and obesity, metabolic syndrome, and insulin resistance [20,21]. However, data from prospective cohort studies suggest that egg intake is not associated with increased risk of coronary heart disease, stroke, and mortality, although it may be associated with increased risk of type 2 diabetes [22,23]. Eggs are an inexpensive food with high quality protein and a rich source of nutrients including choline, folate, selenium, and vitamins A, B, D, E, and K, and, if fortified, ω-3 fatty acids [24]. The nutritional value of eggs can be an important contributor to the health of women of reproductive age and to positive pregnancy outcomes particularly in disadvantaged populations with limited access to more costly healthy foods [24,25]. Folate, choline and docosahexaenoic acid (DHA), all of which can be supplied by eggs, are important nutrients during reproductive age for their role in the prevention of adverse pregnancy outcomes related to fetal central nervous system development [25,26,27].

The World Agricultural Supply and Demand Estimates Report [28] shows that the U.S. had an annual per capita consumption of 263 eggs in 2014, while Mexico is reported to have the highest consumption in the Americas with 335 eggs per person/year. Data from the 2001–2002 National Health and Nutritional Examination Survey (NHANES) suggested that eggs accounted for 7% of the servings from meat and beans group among the general U.S. population [29]. Estimates using NHANES III data suggested that 18.7% of U.S. adults were egg consumers (*i.e.*, individuals who reported intake of at least one egg-group product in a 24-h recall) [30]. Compared to non-consumers, egg consumers were less likely to have inadequate intakes of vitamins B12, A, E, and C. Furthermore, relative to other ethnic groups, a greater proportion of Mexican Americans (31.8%) were egg consumers [30].

Despite the known nutritional content of eggs and their potential benefit towards the health of reproductive-age women, information regarding their contribution to a healthful diet among Hispanic women of reproductive age is limited to a report with a diverse sample of pregnant women, most of them of Caribbean descent [31]. Thus, the objective of the present study was to compare nutrient intake and dietary quality, as assessed by the Healthy Eating Index 2010 (HEI-2010) [32], an algorithm that measures conformance to the 2010 Dietary Guidelines for Americans [33], between egg-consumers and non-consumers among Mexican-American postpartum women.

## 2. Study Design

### 2.1. Participants

Participants were 139 habitually sedentary overweight or obese (BMI between 25 and 35 kg/m^2^) Mexican-American postpartum women (18–40 years old, at least six weeks but less than six months after childbirth) enrolled into *Madres para la Salud* (Mothers for Health; *Madres*), a social support community-based intervention promoting physical activity [34]. Exclusion criteria included currently engaged in regular, strenuous physical activity; currently pregnant or planning on becoming pregnant within the next 12 months; using antidepressants or anti-inflammatory medications; and having a BMI less than 25 or greater than 35 kg/m^2^. Only baseline data were used for the current analysis. The study was approved by Arizona State University’s Institutional Review Board, and all participants provided written consent to participate. The study was registered at ClinicalTrials.gov (Identifier: NCT01908959).

### 2.2. Measures

Trained bilingual research staff collected and processed all baseline sociodemographic, anthropometric and diet data prior to the randomization allocation. Standard procedures were used to measure in triplicate height, weight, and waist circumference.

Three unannounced 24-h recalls using a five-step, multiple-pass method were collected to assess baseline dietary intake data [35]. The dietary recalls were randomly collected during all seven days of the week, including two week days and one weekend day. The Nutrition Data System for Research (NDSR) software version 2009, developed by the Nutrition Coordinating Center (NCC), University of Minnesota, Minneapolis, MN, was used to analyze dietary data. Dietary variables of interest were estimates of total energy intake, amount and percentage of energy provided by macronutrients, and selected micronutrients. Egg intake was reported as servings per day, with one serving being equivalent to one large egg. Participants were classified as egg consumers if they reported any egg intake (including egg products) in at least one of the three 24-h dietary recalls (mean egg intake >0 servings/day).

Diet quality was assessed by calculating the HEI-2010, as described elsewhere [32]. Briefly, the HEI-2010 includes 12 components divided into adequacy components that should be included in the diet (total fruit, whole fruit, total vegetables, greens and beans, whole grains, dairy, total protein foods, seafood and plant proteins, and fatty acids) and moderation components that should be limited (refined grains, sodium, and empty calories). Individual components are scored from 0 to 5, 10, or 20 points; the total HEI-2010 score is calculated as the sum of all individual components, for a total of 100 possible points [36]. Maximum scores correspond to conformance to the dietary guidelines, reflecting higher consumption of adequacy components and lower consumption of moderation components. The scoring standards are assessed as food group and nutrients consumed per intake of 4184 kJ (1000 kcal), percentage of total energy intake, or a ratio, providing information on a density basis rather than absolute amounts [32]. The recommended approach to calculating HEI scores using NDSR was followed [37].

### 2.3. Statistical Analysis

All statistical analyses were conducted with software IBM SPSS Statistics for Windows, version 21.0 (IBM Corp., Armonk, NY, USA). Descriptive characteristics of participants and dietary data are presented in text and tables as mean ± standard deviation (SD). Nutrient intake for carbohydrates, protein, fat, and saturated fat was expressed as percentages of total energy. We used independent groups *t*-tests to compare egg consumers and non-consumers on each of the nutrient intake measures, each HEI-2010 component, and the HEI-2010 total score.

## 3. Results

### 3.1. Participant Characteristics

Participants were 139 women (28 ± 6 years old) self-identified as Mexican or Mexican-American (Table 1). Per study design, all participants were overweight or obese (mean BMI = 29.7 ± 3.5 kg/m^2^) with mean waist and hip circumferences of 86 ± 9 cm and 106 ± 8 cm, respectively, and mean body fat of 38.6% ± 4.6%. Based on dietary intake data from three dietary recalls, 57 women (41% of participants) were classified as egg non-consumers, whereas 82 women (59% of participants) were classified as egg consumers. There were no significant differences in age, BMI, waist or hip circumferences, or body fat percent between egg consumers and non-consumers.

**Table 1:** Characteristics of study participants ^a^.

Characteristics	All (*n* = 139)	Egg Non-Consumers (*n* = 57)	Egg Consumers (*n* = 82)	*p* Value
Age (year)	28.3 ± 5.6	28.8 ± 5.4	27.9 ± 5.7	0.331
Body mass index (kg/m^2^)	29.7 ± 3.5	29.3 ± 3.3	29.9 ± 3.7	0.321
Waist circumference (cm)	86.0 ± 9.0	86.1 ± 9.6	85.9 ± 8.7	0.895
Hip circumference (cm)	105.5 ± 7.6	105.4 ± 7.5	105.5 ± 7.8	0.934
Body fat (%)	38.6 ± 4.6	38.6 ± 4.7	38.5 ± 4.6	0.963

^a^ Data shown as mean ± SD.

### 3.2. Nutrient Intake

Intake of total energy, macronutrient, and select micronutrient intake data for egg consumers and non-consumers are displayed in Table 2. Relative to non-consumers, egg consumers had 14% greater energy intake (*p* = 0.033), associated with consuming 17% more protein (*p* = 0.031) and 19% more fat (*p* = 0.039). There were no significant differences between egg non-consumers and consumers in the absolute amount of saturated and polyunsaturated fat, including ω-3 fatty acids, but monounsaturated fat intake was 24% greater for egg consumers than non-consumers (*p* = 0.020). There were no significant differences between egg non-consumers and consumers in the proportion of energy provided by macronutrients (data not shown), with the exception of monounsaturated fat (10.5% ± 2.4% of energy for non-consumers *vs.* 11.6% ± 3.1% of energy for consumers; *p* = 0.026). As expected, egg consumers had greater intake of dietary cholesterol (109%; *p* < 0.0001) than non-consumers. Whereas there were no significant differences between groups in total or added sugars intake, egg consumers had greater intake of total fiber (22%; *p* = 0.035) and soluble fiber (29%; *p* = 0.017) relative to non-consumers.

Regarding micronutrient intake (Table 2), there were no significant differences between groups in dietary vitamin A, vitamin C, vitamin E, vitamin K, thiamin, niacin, vitamin B6, folate, calcium, or iron. However, egg consumers had greater intakes of vitamin D (31%; *p* = 0.033), riboflavin (29%; *p* = 0.006), vitamin B12 (32%; *p* = 0.031), choline (59%; *p* < 0.0001), sodium (23%; *p* = 0.008), potassium (21%; *p* = 0.011), and phosphorus (19%; *p* = 0.012) than non-consumers. There were no significant differences in dietary lutein + zeaxanthin between groups.

**Table 2:** Comparison of total energy, macronutrient, and select micronutrient intake between egg consumers and egg non-consumers among postpartum Mexican American adult women ^a^.

Nutrient	DRI ^b^	All (*n* = 139)	Egg Non-Consumers (*n* = 57)	Egg Consumers (*n* = 81)	*p* Value
Energy (kJ)		6137 ± 2208	5660 ± 1934	6468 ± 2334	0.033
Energy (kcal)		1466 ± 528	1353 ± 462	1546 ± 561	0.033
Total Carbohydrate (g)	160	195 ± 69	184 ± 61	203 ± 74	0.113
Total Protein (g)	0.66/kg	59 ± 24	53 ± 22	62 ± 25	0.031
Total Fat (g)		52 ± 25	47 ± 24	56 ± 26	0.039
Saturated Fat (g)		18 ± 9	16 ± 8	19 ± 9	0.131
Monounsaturated Fat (g)		19 ± 10	17 ± 9	21 ± 10	0.020
Polyunsaturated Fat (g)		11 ± 6	10 ± 7	12 ± 6	0.138
ω-3 Fatty Acids (g)		1.09 ± 0.77	0.99 ± 0.83	1.15 ± 0.71	0.205
Cholesterol (mg)		228 ± 148	139 ± 124	291 ± 131	0.0001
Total sugars (g)		93.7 ± 41.3	88.4 ± 37.2	97.3 ± 43.8	0.213
Added sugars (g)		62.9 ± 35.3	61.6 ± 33.4	63.8 ± 36.7	0.715
Total dietary fiber (g)	25	13.5 ± 7.0	12.0 ± 5.0	14.6 ± 8.0	0.035
Soluble fiber (g)		4.1 ± 2.6	3.5 ± 1.5	4.5 ± 3.1	0.017
Vitamin A (μg) ^c^	500	625 ± 396	563 ± 401	668 ± 389	0.126
Vitamin C (mg)	60	62.3 ± 53.8	54.0 ± 44.2	68.2 ± 59.2	0.128
Vitamin D (μg)	10	4.95 ± 3.53	4.19 ± 3.39	5.48 ± 3.55	0.033
Vitamin E (mg)	12	4.92 ± 3.02	4.48 ± 3.21	5.22 ± 2.87	0.159
Vitamin K (μg)	90	42.5 ± 42.6	38.6 ± 34.8	45.2 ± 47.2	0.375
Thiamin (mg)	0.9	1.22 ± 0.57	1.16 ± 0.54	1.26 ± 0.58	0.268
Riboflavin (mg)	0.9	1.67 ± 0.86	1.43 ± 0.76	1.83 ± 0.90	0.006
Niacin (mg)	11	16.7 ± 7.9	15.9 ± 7.9	17.3 ± 8.0	0.328
Vitamin B6 (mg)	1.1	1.60 ± 1.09	1.44 ± 1.00	1.70 ± 1.14	0.165
Total folate (μg)	320	332 ± 210	295 ± 197	357 ± 217	0.090
Vitamin B12 (μg)	2.0	4.74 ± 3.48	3.98 ± 3.00	5.27 ± 3.70	0.031
Choline (mg)	425	237 ± 110	176 ± 79	279 ± 110	0.0001
Sodium (mg)	1500	2569 ± 1132	2266 ± 945	2780 ± 1206	0.008
Potassium (mg)	4700	1788 ± 781	1587 ± 629	1927 ± 848	0.011
Calcium (mg)	800	779 ± 358	735 ± 301	826 ± 390	0.142
Phosphorus (mg)	580	1018 ± 415	913 ± 349	1091 ± 443	0.012
Iron (mg)	8.1	12.6 ± 7.0	11.4 ± 6.4	13.4 ± 7.3	0.091
Lutein + Zeaxanthin (μg)		680 ± 979	547 ± 648	773 ± 1150	0.181

^a^ Data shown as mean ± SD; ^b^ DRI = Dietary Reference Intake for women 31–50 years old; displayed as Adequate Intake for fiber, choline, vitamin K, sodium and potassium, and as Estimated Average Requirement for all other nutrients, when applicable; ^c^ Retinol Equivalents.

### 3.3. Healthy Eating Index 2010

Estimated mean HEI-2010 individual component and total scores for egg non-consumers and consumers are presented in Table 3. There were no significant differences in adequacy component scores between groups, with the exception of the total protein foods score, which was greater in egg consumers than in non-consumers (*p* = 0.004). There was a non-significant trend for egg consumers to have a greater total fruit score relative to non-consumers (*p* = 0.070). There were no significant differences between groups in moderation component scores, although relative to non-consumers, there were trends for egg consumers to have a lower score (greater intake) for sodium (score of 4.5 ± 3.5 *vs.* 3.5 ± 3.0 out of 10; *p* = 0.074) and a greater score (lower intake) for empty calories (score of 8.8 ± 5.7 *vs.* 10.6 ± 5.3 out of 20; *p* = 0.059). The total composite HEI-2010 score did not differ significantly between groups, although was slightly greater for egg consumers (52 ± 14 *vs.* 56 ± 13 out of 100 points for egg non-consumers and consumers, respectively; *p* = 0.082).

**Table 3:** Estimated mean total Healthy Eating Index (HEI)-2010 and component scores among between egg consumers and egg non-consumers among postpartum Mexican American adult women, expressed as absolute score and percentage of the maximum score ^a^.

Component (Score Range)	All (*n* = 139)	Egg Non-Consumers (*n* = 57)	Egg Consumers (*n* = 82)	*p* Value
*Adequacy Components*				
Total Fruit (0–5)	2.2 ± 1.7	1.9 ± 1.7	2.4 ± 1.8	0.070
Whole Fruit (0–5)	2.4 ± 1.9	2.1 ± 1.9	2.6 ± 1.9	0.168
Total Vegetables (0–5)	2.5 ± 1.2	2.6 ± 1.3	2.5 ± 1.1	0.710
Greens and Beans (0–5)	1.6 ± 1.6	1.8 ± 1.9	1.4 ± 1.4	0.227
Whole Grains (0–10)	7.6 ± 3.2	7.6 ± 3.4	7.6 ± 3.1	0.885
Dairy (0–10)	5.9 ± 3.3	5.8 ± 3.3	6.0 ± 3.3	0.729
Total Protein Foods (0–5)	4.5 ± 0.9	4.3 ± 1.0	4.7 ± 0.7	0.004
Seafood and Plant Proteins (0–5)	2.8 ± 2.1	2.2 ± 2.1	2.7 ± 2.1	0.173
Fatty Acids (0–0)	4.3 ± 2.9	3.9 ± 2.8	4.6 ± 3.0	0.116
*Moderation Components*				
Refined Grains (0–10)	7.5 ± 3.1	7.0 ± 3.2	7.8 ± 3.1	0.127
Sodium (0–10)	3.9 ± 3.3	4.5 ± 3.5	3.5 ± 3.0	0.074
Empty Calories (0–20)	9.8 ± 5.5	8.8 ± 5.7	10.6 ± 5.3	0.059
*Total Score* (0–100)	54.7 ± 13.5	52.3 ± 14.4	56.4 ± 12.6	0.082

^a^ Data shown as mean ± SD.

## 4. Discussion

This study assessed the contribution of eggs to dietary quality among an understudied group of the population, Mexican-American postpartum women, by comparing nutrient intake and HEI-2010 scores between egg-consumers and non-consumers. This report is of importance because there is limited information regarding contributors to nutrient intake and dietary quality among Mexican-American women of reproductive age. Study findings indicated that egg consumers had greater intakes of energy, protein, fat, monounsaturated fat, cholesterol, total and soluble fiber, vitamin D, riboflavin, vitamin B12, choline, sodium, potassium, and phosphorus, and a modestly higher HEI-2010 score than non-consumers. Similarly, in the only study of its kind conducted in a diverse sample of pregnant Hispanic women, egg consumers had higher intakes of several nutrients including protein, fat, vitamins K and E, cholesterol, total polyunsaturated fatty acids, and DHA [31]. Comparable information among postpartum women is lacking.

In the current study, a greater proportion of participants (59% or *n* = 82) reported consuming eggs than what has been reported for U.S. adults (19%) or Mexican-American adults (32%) using NHANES III data [30]. A majority of study participants (78%) were immigrants of Mexican origin who had been in the U.S. for 12 ± 7 years [34,38,39]. Several reports indicate that multiple factors that are part of the immigration experience, such as acculturation, generational status, and time in the U.S., are associated with lifestyle modifications often associated with less healthful dietary patterns in part due to immigrants’ lack of familiarity with available foods in their new environment [40,41,42,43]. However, eggs are a low-cost common protein source in the Mexican diet, as evidenced by the high per capita consumption of eggs in Mexico compared to other countries [28], which may have made eggs a nutritious and familiar dietary choice among the generally low-socioeconomic status study participants [34,38,39]. Nevertheless, the possibility cannot be ruled out that the methodology used in the current study (three 24-h recalls) may have better allowed to capture individual instances of egg intake than using only one 24-h recall for NHANES.

In the current study, energy intake was greater for egg consumers than non-consumers. Considering that participants were overweight or obese, this increased energy intake could be of concern because it could potentially translate into long-term weight gain, even when mean total energy intake was lower than what has been reported among U.S. adult women (7543 ± 59 kJ/day (1803 ± 14 kcal/day) according to NHANES 2009-2010 data [44]). The greater energy intake among egg consumers could have been in part attributed to greater fat intake. Differences in fatty acid intake were due to greater monounsaturated fatty acids in the diet of egg consumers *vs.* non-consumers, which explains the greater, albeit non-significant, HEI-2010 fatty acid ratio score ((polyunsaturated fatty acids + monounsaturated fatty acids)/saturated fatty acids) for egg consumers. Although these differences cannot be solely attributed to the fatty acid contribution from eggs, it is noteworthy that roughly 50% of fatty acids in eggs are monounsaturated [45]. Regarding polyunsaturated fatty acids, fortified eggs can be an important dietary contributor to ω-3 fatty acids. A study including Mexican pregnant women reported that 20% of total DHA intake was supplied by eggs [27]. In a diverse sample of pregnant Hispanic women, a greater proportion of egg consumers than non-consumers had DHA consumption in the highest tertile of intake [31]. In the current study, differences in ω-3 fatty acid intake between egg non-consumers and consumers were not statistically significant. Although as expected in the current study egg consumers had greater dietary cholesterol than non-consumers, intake levels did not exceed current recommendations for cholesterol intake (300 mg/day) [46].

Adequate protein intake is crucial for women of reproductive age to maintain maternal and fetal tissue accretion during pregnancy and milk production during lactation [47,48,49]. In fact, a recent report [50] suggested that protein needs for pregnancy may be even higher than current dietary reference intake recommendations of 1.1 g/kg of body weight/day [46]. In the current study, both absolute protein intake and the HEI-2010 score for total protein foods were greater for egg consumers than non-consumers, although as previously reported the total protein HEI-2010 score was indicative of appropriate consumption of protein-containing foods for all participants [38]. Intake of animal protein was also greater for egg consumers than for non-consumers, although non-significantly different (data not shown). However, the high-quality nature of egg protein [24] may provide an additional nutritional advantage to women who incorporate eggs as part of their diet in the postpartum period.

In the present study, egg-consumers had a greater intake of vitamin D compared to non-consumers. According to NHANES 2003–2006 data, eggs were ranked the third main source of vitamin D (after milk and fish/seafood), contributing with about 5% of vitamin D intake among adults in the United States [51]. This is important considering that it is estimated that between 40% and 80% of U.S. adults, depending on gender and age range, have inadequate vitamin D intake [52]. Furthermore, results from a meta-analysis study showed that low maternal vitamin D levels during pregnancy are associated with risk of preeclampsia, gestational diabetes, preterm birth and small-for-gestational age [53].

All participants in the current study had HEI-2010 scores suggesting intake of diets with low quality or in need for improvement (55 ± 14 relative to a maximum score of 100). A report using NHANES data suggested that at the population level HEI-2010 total score was 53 [54]. Furthermore, participants were not meeting intake recommendations for several nutrients, including fiber, Ca, vitamin E, vitamin C and folate. Egg consumers had a greater HEI-2010 score for total protein foods, and trends towards greater scores for total fruit and empty calories, and lower score for sodium. These differences resulted in a modestly greater total HEI-2010 score for egg consumers.

The main risk associated with egg consumption in the U.S. is salmonellosis, mainly due to consumption of raw or undercooked eggs [55]. The risk is particularly important during pregnancy since salmonellosis may be transmitted to the fetus, increasing the risk of preterm delivery and intrauterine death [55,56]. In a study about food safety, most pregnant women were aware about recommendations to avoid raw eggs; however, they still reported consumption of eggs with runny yolk (35%) and cookie dough containing raw eggs (40%), indicating the need to improve instructions given to this target group [57]. Nevertheless, the potential benefits associated with their high nutritional quality outweigh risks as long as eggs are properly cooked before consumption.

Prior reports from the current study have documented that participants included a large proportion of women living under disadvantaged socio-economic conditions in neighborhoods with limited food access [34,38,39]. In general, Hispanic households have been documented to have greater food insecurity rates than the general population [58]. Thus, an additional benefit of egg intake for the target population in the current study as well as other Hispanic subgroups is associated with their low cost. In general, the cost of foods is directly associated with protein but inversely associated with carbohydrate content [59]. In contrast, relative to other animal sources of protein, eggs are an inexpensive nutrient-dense, high-protein, low-carbohydrate food [24,59]. According to the Nutrient Rich Foods Index, developed as an effort to aid consumers select nutritious food choices under financial constraints, eggs are a low cost source of protein, vitamin A, vitamin B12, riboflavin, calcium and zinc, providing excellent nutritional value for the money [60].

An important limitation of the current report is that dietary assessment was conducted using three 24-h dietary recalls, a method subject to intake underreporting, particularly among women and Hispanic individuals [61,62], as indicated by the relatively low energy intake observed in the current study. Although this limitation was addressed by calculating the HEI-2010 scores on an energy-density basis when assessing dietary quality [32], differences observed in individual nutrient intakes between egg consumers and non-consumers, particularly macronutrients, are likely a result of absolute energy intake and not solely due to egg consumption. Moreover, data from 24-h dietary recalls may not be the best indicator of habitual food consumption, including eggs.

## 5. Conclusions

The current report suggests that Mexican-American women who consumed eggs had greater nutrient intake and higher dietary quality than non-consumers. Whereas these data do not indicate that egg intake directly improves dietary quality, it could potentially be an indicator of a healthier dietary pattern. Despite the greater energy intake observed among egg consumers, the low cost and high nutritional quality of eggs make them an ideal protein source that is likely to provide greater benefit than risk to the target population, especially if dietary recommendations focus on consuming eggs as part of a dietary pattern that also includes nutrient-dense and fiber-rich foods and maintaining a healthy body weight.

## References

[1] Colby S.L., Ortman J.M., U.S. Census Bureau (2014). Projections of the Size and Composition of the U.S. Population: 2014 to 2060.

[2] Pan L., Galuska D.A., Sherry B., Hunter A.S., Rutledge G.E., Dietz W.H., Balluz L.S. (2009). Differences in Prevalence of Obesity among Black, White and Hispanic Adults—United States, 2006–2008.

[3] Daviglus M.L., Talavera G.A., Avilés-Santa M.L., Allison M., Cai J., Cirqui M.H., Gellman M., Giachello A.L., Gouskova N., Kaplan R.C. (2012). Prevalence of major cardiovascular risk factors and cardiovascular diseases among Hispanic/Latino individuals of diverse backgrounds in the United States. JAMA.

[4] Rooney B.L., Schauberger C.W., Mathiason M.A. (2005). Impact of perinatal weight change on long-term obesity and obesity-related illnesses. Obstet. Gynecol..

[5] Ogden C.L., Carroll M.D., Curtin L.R., McDowell M.A., Tabak C.J., Flegal K.M. (2006). Prevalence of overweight and obesity in the United States, 1999–2004. JAMA.

[6] Records K., Keller C., Ainsworth B., Permana P.A. (2008). Overweight and obesity in postpartum Hispanic women. Health Care Women Int..

[7] Chiuve S.E., Fung T.T., Rimm E.B., Hu F.B., McCullough M.L., Wang M., Stampfer M.J., Willett W.C. (2012). Alternative dietary indices both strongly predict risk of chronic disease. J. Nutr..

[8] Nicklas T.A., O’Neil C.E., Fulgoni V.L. (2012). Diet quality is inversely related to cardiovascular risk factors in adults. J. Nutr..

[9] Carrera P.M., Gao X., Tucker K.L. (2007). A study of dietary patterns in the Mexican-American population and their association with obesity. J. Am. Diet. Assoc..

[10] Ervin R.B., Ogden C.L. (2013). Consumption of Added Sugars among U.S. Adults, 2005–2010.

[11] Kirkpatrick S.I., Dodd K.W., Reedy J., Krebs-Smith S.M. (2012). Income and race/ethnicity are associated with adherence to food-based dietary guidance among US adults and children. J. Acad. Nutr. Diet..

[12] Ogden C.L., Kit B.K., Carroll M.D., Park S. (2011). Consumption of Sugar Drinks in the United States, 2005–2008.

[13] Hoerr S.L., Tsuei E., Liu Y., Franklin F.A., Nicklas T.A. (2008). Diet quality varies by race/ethnicity of Head Start mothers. J. Am. Diet. Assoc..

[14] Lichtenstein A.H., Appel L.J., Brands M., Carnethon M., Daniels S., Franch H.A., Franklin B., Kris-Etherton P., Harris W.S., Howard B. (2006). Diet and lifestyle recommendations revision 2006. A scientific statement from the American Heart Association Nutrition Committee. Circulation.

[15] Spence J.D., Jenkins D.J.A., Davignon J. (2010). Dietary cholesterol and egg yolks: Not for patients at risk of vascular disease. Can. J. Cardiol..

[16] Fernandez M.L. (2012). Rethinking dietary cholesterol. Curr. Opin. Clin. Nutr. Metab. Care.

[17] Fernandez M.L. (2010). Effects of eggs on plasma lipoproteins in healthy populations. Food Funct..

[18] Hsiao P.Y., Mitchell D.C., Coffman D.L., Allman R.M., Locher J.L., Sawyer P., Jensen G.L., Hartman T.J. (2013). Dietary patterns and diet quality among diverse older adults: The University of Alabama at Birmingham study of aging. J. Nutr. Health Aging.

[19] Bouchard-Mercier A., Paradis A.M., Godin G., Lamarche B., Perusse L., Vohl M.C. (2010). Associations between dietary patterns and LDL peak particle diameter: A cross-sectional study. J. Am. Coll. Nutr..

[20] Flores M., Macias N., Rivera M., Lozada A., Barquera S., Rivera-Dommarco J., Tucker K.L. (2010). Dietary patterns in Mexican adults are associated with risk of being overweight or obese. J. Nutr..

[21] Amini M., Esmaillzadeh A., Shafaeizadeh S., Behrooz J., Zare M. (2010). Relationship between major dietary patterns and metabolic syndrome among individuals with impaired glucose tolerance. Nutrition.

[22] Rong Y., Chen L., Zhu T., Song Y., Yu M., Shan Z., Sands A., Hu F.B., Liu L. (2013). Egg consumption and risk of coronary heart disease and stroke: Dose-response meta-analysis of prospective cohort studies. BMJ.

[23] Shin J.Y., Xun P., Nakamura Y., He K. (2013). Egg consumption in relation to risk of cardiovascular disease and diabetes: A systematic review and meta-analysis. Am. J. Clin. Nutr..

[24] Applegate E. (2000). Introduction: nutritional and functional roles of eggs in the diet. J. Am. Coll. Nutr..

[25] Iannotti L.L., Lutter C.K., Bunn D.A., Stewart C.P. (2014). Eggs: The uncracked potential for improving maternal and young child nutrition among the world’s poor. Nutr. Rev..

[26] Caudill M.A. (2010). Pre- and postnatal health: Evidence of increased choline needs. J. Am. Diet. Assoc..

[27] Parra-Cabrera S., Stein A.D., Wang M., Martorell R., Rivera J., Ramakrishnan U. (2011). Dietary intakes of polyunsaturated fatty acids among pregnant Mexican women. Mater. Child Nutr..

[28] U.S. Department of Agriculture (2015). World Agricultural Supply and Demand Estimates.

[29] Bachman J.L., Reedy J., Subar A.F., Krebs-Smith S.M. (2008). Sources of food group intakes among the US population, 2001–2002. J. Am. Diet. Assoc..

[30] Song W.O., Kerver J.M. (2000). Nutritional contribution of eggs to American diets. J. Am. Coll. Nutr..

[31] Bermudez-Millán A., Hromi-Fiedler A., Damio G., Segura-Perez S., Perez-Escamilla R. (2009). Egg contribution towards the diet of pregnant Latinas. Ecol. Food Nutr..

[32] Guenther P.M., Casavale K.O., Reedy J., Kirkpatrick S.I., Hiza H.A., Kuczynski K.J., Kahle L.L., Krebs-Smith S.M. (2013). Update of the Healthy Eating Index: HEI-2010. J. Acad. Nutr. Diet..

[33] U.S. Department of Agriculture, U.S. Department of Health and Human Services (2010). Dietary Guidelines for Americans, 2010.

[34] Keller C.S., Records K., Ainsworth B.E., Belyea M., Permana P.A., Coonrod D.V., Vega-López S., Nagle-Williams A. (2011). Madres para la Salud: Design of a theory-based intervention for postpartum Latinas. Contemp. Clin. Trials.

[35] Conway J.M., Ingwersen L.A., Moshfegh A.J. (2004). Accuracy of dietary recall using the USDA five-step multiple-pass method in men: An observational validation study. J. Am. Diet. Assoc..

[36] Freedman L.S., Guenther P.M., Krebs-Smith S.M., Kott P.S. (2008). A population’s mean Healthy Eating Index-2005 scores are best estimated by the score of the population ratio when one 24-hour recall is available. J. Nutr..

[37] Miller P.E., Mitchell D.C., Harala P.L., Pettit J.M., Smiciklas-Wright H., Hartman T.J. (2011). Development and evaluation of a method for calculating the Healthy Eating Index-2005 using the Nutrition Data System for Research. Public Health Nutr..

[38] Pignotti G.A., Vega-López S., Keller C., Belyea M., Ainsworth B., Nagle Williams A., Records K., Coonrod D., Permana P. (2015). Comparison and evaluation of dietary quality between older and younger Mexican-American women. Public Health Nutr..

[39] Keller C., Todd M., Ainsworth B., Records K., Vega-Lopez S., Permana P., Coonrod D., Nagle Williams A. (2013). Overweight, obesity, and neighborhood characteristics among postpartum Latinas. J. Obes..

[40] Ayala G.X., Baquero B., Klinger S. (2008). A systematic review of the relationship between acculturation and diet among Latinos in the United States: Implications for future research. J. Am. Diet. Assoc..

[41] Himmelgreen D., Romero Daza N., Cooper E., Martinez D. (2007). “I don’t make the soups anymoreˮ: Pre- to post-migration dietary and lifestyle chanes among Latinos living in West-Central Florida. Ecol. Food Nutr..

[42] Guendelman S., Abrams B. (1995). Dietary intake among Mexican-American women: Generational differences and a comparison with white non-Hispanic women. Am. J. Public Health.

[43] Perez-Escamilla R. (2011). Acculturation, nutrition, and health disparities in Latinos. Am. J. Clin. Nutr..

[44] Ford E.S., Dietz W.H. (2013). Trends in energy intake among adults in the United States: Findings from NHANES. Am. J. Clin. Nutr..

[45] Samman S., Kung F.P., Carter L.M., Foster M.J., Ahmad Z.I., Phuyal J.L., Petocz P. (2009). Fatty acid composition of certified organic, conventional and omega-3 eggs. Food Chem..

[46] Institute of Medicine (2005). Dietary Reference Intakes for Energy, Carbohydrate, Fiber, Fat, Fatty Acids, Cholesterol, Protein, and Amino Acids (Macronutrients).

[47] King J.C. (2000). Physiology of pregnancy and nutrient metabolism. Am. J. Clin. Nutr..

[48] Kalhan S.C. (2000). Protein metabolism in pregnancy. Am. J. Clin. Nutr..

[49] Manjarin R., Bequette B.J., Wu G., Trottier N.L. (2014). Linking our understanding of mammary gland metabolism to amino acid nutrition. Amino Acids.

[50] Stephens T.V., Payne M., Ball R.O., Pencharz P.B., Elango R. (2015). Protein requirements of healthy pregnant women during early and late gestation are higher than current recommendations. J. Nutr..

[51] O’Neil C.E., Keast D.R., Fulgoni V.L., Nicklas T.A. (2012). Food sources of energy and nutrients among adults in the US: NHANES 2003–2006. Nutrients.

[52] Bailey R.L., Dodd K.W., Goldman J.A., Gahche J.J., Dwyer J.T., Moshfegh A.J., Sempos C.T., Picciano M.F. (2010). Estimation of total usual calcium and vitamin D intakes in the United States. J. Nutr..

[53] Wei S.Q., Qi H.P., Luo Z.C., Fraser W.D. (2013). Maternal vitamin D status and adverse pregnancy outcomes: A systematic review and meta-analysis. J. Mater.Fetal Neonatal Med..

[54] Guenther P.M., Casavale K.O., Kirkpatrick S.I., Reedy J., Hiza H.A.B., Kuczynski K.J., Kahle L.L., Krebs-Smith S.M. (2013). Diet Quality of Americans in 2001–02 and 2007–08 as Measured by the Healthy Eating Index-2010.

[55] Voetsch A.C., van Gilder T.J., Angulo F.J., Farley M.M., Shallow S., Marcus R., Cieslak P.R., Deneen V.C., Tauxe R.V., Emerging infections program FoodNet working group (2004). FoodNet estimate of the burden of illness caused by nontyphoidal Salmonella infections in the United States. Clin. Infect. Dis..

[56] Kaiser L., Allen L.H. (2008). Position of the American Dietetic Association: Nutrition and lifestyle for a healthy pregnancy outcome. J. Am. Diet. Assoc..

[57] Athearn P.N., Kendall P.A., Hillers V.V., Schroeder M., Bergmann V., Chen G., Medeiros L.C. (2004). Awareness and acceptance of current food safety recommendations during pregnancy. Matern. Child Health J..

[58] Coleman-Jensen A., Gregory C., Singh A. (2014). Household Food Security in the United States in 2013.

[59] Brooks R.C., Simpson S.J., Raubenheimer D. (2010). The price of protein: Combining evolutionary and economic analysis to understand excessive energy consumption. Obes. Rev..

[60] Drewnowski A. (2010). The Nutrient Rich Foods Index helps to identify healthy, affordable foods. Am. J. Clin. Nutr..

[61] Neuhouser M.L., Tinker L., Shaw P.A., Schoeller D., Bingham S.A., Horn L.V., Beresford S.A., Caan B., Thomson C., Satterfield S. (2008). Use of recovery biomarkers to calibrate nutrient consumption self-reports in the Women’s Health Initiative. Am. J. Epidemiol..

[62] Bothwell E.K.G., Ayala G.X., Conway T.L., Rock C.L., Gallo L.C., Elder J.P. (2009). Underreporting of food intake among Mexican/Mexican-American women: Rates and correlates. J. Am. Diet. Assoc..

